# Nutrition for Gestational Diabetes—Progress and Potential

**DOI:** 10.3390/nu12092685

**Published:** 2020-09-03

**Authors:** Clive J. Petry

**Affiliations:** Department of Paediatrics, Cambridge Biomedical Campus, University of Cambridge, Box 116, Cambridge CB2 0QQ, UK; cjp1002@cam.ac.uk; Tel.: +44-(0)1223-762-945

Gestational diabetes (GDM), traditionally defined as any form of glucose intolerance first detected in pregnancy [1], still has dietary treatment as its frontline therapy [2]. Whilst some women require additional pharmacotherapy, such as with insulin or metformin, nutritional intake is relevant to all women with GDM. Recognition of the importance of GDM is growing due to the worldwide increase in its prevalence [3], rising in line with the increased prevalence of overweight and obesity [4], and the impact of its short- and long-term complications in both the mother and offspring exposed to GDM in utero [5]. In fact, it has been suggested that GDM makes a significant contribution to the current diabetes epidemic [6]. This partially relates to the increased risk of obesity [7], insulin resistance [8], GDM [9] and type 2 diabetes [10] in people who were exposed to GDM in fetal life, showing that “diabetes begets diabetes” [11]. Thus, preventing and treating GDM more efficiently have a sense of urgency about them, with nutritional modifications needing to be at the forefront. This is especially since a recent keynote systematic review and meta-analysis of nutritional interventions in GDM that were tested in randomized, controlled trials found favorable effects on maternal glycemic control and neonatal growth parameters when grouped together [12]. It is with these considerations that this Special Issue of Nutrients has been published, looking at nutrients or factors related to nutrition that may be involved either in the prevention, development or treatment of GDM. In this way, I hope that it plays some part in showing current progress and the potential for future improvements in nutrition for GDM.

Whilst the majority of papers in this Special Issue related more to the treatment or complications of GDM once diagnosed, there are several that related more to the development or the prevention of GDM. Mitanchez and colleagues [13] presented a systematic review of recent meta-analyses relating to the effects of maternal lifestyle interventions (such as nutritional interventions and participation in specific exercise regimes) on the prevention of GDM (as well as effects on gestational weight gain and neonatal outcomes). They reported that these lifestyle interventions showed a decreased risk for the development of GDM of between 15% and 40% in the meta-analyses. The positive effect in reducing the risk of GDM was greater for exercise than for dietary modification. However, pre-pregnancy biatric surgery led to a reduction in risk for GDM of between 70% and 80% suggesting that lifetime nutrition (and other factors that affect body weight, e.g., exercise) can have a big effect on GDM risk. Another paper in this Special Issue, by Robinson et al. [14], looked at differences in the gut microbiome of overweight and obese women who were ketonuric at week 16 of pregnancy when fasting, matching them to non-ketonuric controls by a number of factors including future GDM status. As well as being an analysis from the Study of Probiotics IN Gestational diabetes (SPRING study), this study is relevant to the development of GDM since ketonuria is common in pregnancy [15] and is associated with consuming a low carbohydrate diet [16], which may be a popular choice for women at high risk of developing GDM in an attempt to control body weight [17]. Robinson et al. [14] found that ketonuric women had an increased abundance of the butyrate-producing genus *Roseburia* in their gut microbiome. Interestingly, one of the gut bacteria spp. that contributed the most to the differences in the composition of the gut microbiota in ketonuric women was *Methanobrevibacter*, which, when assessing metagenomic linkage groups, in a previously published metagenome-wide association study of GDM was found to be enriched in healthy controls [18]. The third paper in this Special Issue, predominantly related to the time in pregnancy before any diagnosis of GDM, was from Cambridge Baby Growth Study investigators [19]. In their contemporary birth and infancy growth cohort they, like many investigators in other studies, had noticed a temporal trend in the incidence of GDM [20]. In their cohort, this temporal trend was associated with an index of deprivation and reduced insulin secretion. Deprivation was not itself directly associated with GDM; however, it was suggested that something related to deprivation was actually mediating the association, such as dietary composition. Using a food frequency questionnaire, with all its inherent limitations [21], they found that the food type most consistently tracking the previously observed trends was eggs. In fact, egg consumption appeared to be protective against GDM, although the effect size was small [19]. Egg consumption in isolation may not totally explain the associations; however, principal component analysis suggested that in this cohort it was positively linked to the consumption of fresh fruit and green vegetables, salad, yogurt and tap water, i.e., a “healthy” diet. In the fourth paper of this type in this Special Issue, Dong et al. [22] found that women that reported the fastest eating speeds early in pregnancy had an increased risk of developing GDM later in pregnancy. The eating speed was strongly related to the pre-pregnancy BMI, however, and adjusting for this attenuated the increased risk for GDM. Assuming that eating speeds did not change following conception, this suggests that increased eating speed may have led to increased weight gain prior to pregnancy and that it was this that led to the increased GDM risk. Consistent with this, fast eating speed has also previously been reported to lead to obesity [23]. Finally, in terms of papers published in this Special Issue related more to the development of GDM than to the period of pregnancy after its diagnosis, Filardi et al. [24] reviewed links between endocrine disrupting chemicals (EDCs) and complications of pregnancy, including the development of GDM. Non-nutritive EDCs extensively pollute the diet and their effects are thought to be linked to those of nutrients through being obesogenic [25]. Potential roles in lowering insulin sensitivity and pancreatic β-cell function are thought to explain the associations between circulating or urinary EDC concentrations and GDM found in some studies [26,27].

GDM is usually formally diagnosed around the start of the third trimester of pregnancy, although effects upon the tempo of fetal growth in women subsequently diagnosed with GDM may already be evident by then [28]. Most body weight is laid down by the fetus in this trimester [29], and the increased body weight observed in babies born to mothers with GDM [30] may at least partially related to increased placental leptin production, as highlighted in the review by Pérez-Pérez [31] in this Special Issue. The good news, however, is that because most fetal body weight is laid down in the third trimester, nutritional adaptations at this point of pregnancy are still potentially able to improve GDM outcomes [12]. Women diagnosed with GDM may be amenable to make lifestyle alterations in order to reduce the risk of GDM-related complications [32]. This is likely to have positive benefits, as the systematic review by Mitanchez and colleagues [13] in this Special Issue reported that combined lifestyle modifications, in terms of a range of dietary adaptations and increased activity levels, lead to reduced fetal growth and neonatal fat mass in women with GDM and lower rates of preterm birth and shoulder dystocia. Atakora and colleagues, also in this Special Issue, presented a secondary analysis of the UK Pregnancies Better Eating and Activity Trial [33] where they reported that a diagnosis of GDM in obese women led to greater reductions in energy and carbohydrate intakes, and glycemic load relative to that of obese pregnant women without GDM. In addition, there was a greater increase in protein consumption. The women with GDM also put on less third trimester body weight. These factors led to lower birth weights of their offspring, although they were born around a week earlier than those babies born to women without GDM. These results suggest that current nutritional strategies to treat women with GDM are effective in promoting behavior change [33], even if the target of normoglycemia has not been routinely achieved yet. Further evidence that women diagnosed with GDM are amenable in making dietary changes come from the Growing Up in New Zealand Study paper by Lawrence and colleagues in this Special Issue [34]. In their cohort, women with GDM reported lower scores for consuming “junk” and “traditional/white bread” dietary patterns and a higher chance of having received dietary advice from a professional. They also had a higher tendency to avoid foods that are high in fat or sugar content.

Not surprisingly for a condition diagnosed according to high circulating glucose concentrations in pregnancy, the bedrock of nutritional therapy for GDM relates to the control of the amount of carbohydrate intake, and to a lesser extent the type of carbohydrate. In this Special Issue, Mustad and colleagues reviewed the role of carbohydrates in the prevention and treatment of GDM [35]. They concluded that concentrating on the amount and type of dietary carbohydrate can have important benefits for GDM pathophysiology, but interventions such as those currently implemented may be inadequate to prevent or treat GDM. Another factor that may be important as regards carbohydrate intake in GDM is timing, as shown by a study by Rasmussen and colleagues in this Special Issue [36]. Using a randomized crossover design, they found that the consumption of a high carbohydrate and energy dietary content in the morning and a low carbohydrate and energy dietary content in the evening in women with GDM (compared to the consumption of a diet with low carbohydrate in the morning and high in the evening) led to lower mean and fasting glucose concentrations and insulin resistance, but higher glucose variability. It is not always easy to predict what circulating glucose concentrations will rise to after the consumption of certain foods, especially those with high carbohydrate contents. Pustozerov and colleagues previously published an algorithm that they used as part of a recommender system infrastructure that incorporates models used to predict circulating glucose concentrations in women with GDM [37] designed, ultimately, to make specific nutritional recommendations to help try and achieve normoglycemia. They found that the prediction of the circulating glucose concentration one hour after eating was not as effective as hoped. So, they did a follow-up study, published in this Special Issue [38], where they incorporated the glycemic index and glycemic load data into their algorithm (both of which have shown relevance to circulating glucose concentrations in pregnancy/GDM [39,40,41]) in an effort to try and improve its prediction accuracy. Unfortunately, whilst there was an improvement in accuracy, it was very modest [38]. Of interest, however, was the finding that the predicted glucose concentrations were correlated more strongly with the glucose load than with the amount of carbohydrate consumed, which the authors suggested could explain the effect of low glycemic index diets in GDM treatment.

The subject matters of the final couple of the papers in this Special Issue [42,43] do not fit neatly into the groups of papers described above. Like all forms of diabetes, GDM is thought to result from a combination of insulin resistance and inadequate insulin secretion [44]. A number of inflammatory biomarkers are thought to stimulate both enhanced insulin resistance [45] and reduced insulin secretion in pregnancy [46], potentially contributing towards the development of GDM or even its complications. In this Special Issue, Piuri and colleagues tracked circulating inflammatory biomarker concentrations in the last trimester of pregnancy in women who were newly diagnosed with GDM which was treated by a (validated) strict adherence to a prescribed diet [42]. They found that circulating concentrations of tumor necrosis factor-α and platelet-activating factor increased over the final 12 weeks of pregnancy, unlike markers of glucose control which were not surprisingly higher than those observed in non-pregnant, healthy women (suggesting that the dietary treatment of GDM was sub-optimal). The authors suggested that these inflammatory biomarkers may therefore contribute to the cause or be a consequence of GDM complications [42]. In the final paper from this Special Issue described in this editorial, Peila and colleagues reviewed one aspect of the influence of GDM after the mother has given birth, that of human breast milk contents [43]. This area is important since it could help to mediate some of the observed transgenerational effects of GDM [7,8,9,10]. The authors observed that GDM appears to be associated with an altered milk composition, although the low number of studies and differences in the types of human milks studied (colostrum, transitional, and mature milk) limit the overall conclusions that can be drawn thus far [43].

This Special Issue covers a wide range of topics related to nutrition for GDM. Whilst progress has been made in both dietary means of reducing the risk of developing GDM in high risk women who are either already pregnant or planning to get pregnant, and in dietary treatment for GDM, there is clearly progress still to be made. As well as emphasizing current research in this area, hopefully this Special Issue also highlights gaps in the knowledge that need to be filled in future research studies.
